# The β_2_ clamp in the *Mycobacterium tuberculosis* DNA polymerase III αβ_2_ε replicase promotes polymerization and reduces exonuclease activity

**DOI:** 10.1038/srep18418

**Published:** 2016-01-29

**Authors:** Shoujin Gu, Wenjuan Li, Hongtai Zhang, Joy Fleming, Weiqiang Yang, Shihua Wang, Wenjing Wei, Jie Zhou, Guofeng Zhu, Jiaoyu Deng, Jian Hou, Ying Zhou, Shiqiang Lin, Xian-En Zhang, Lijun Bi

**Affiliations:** 1 Key Laboratory of RNA Biology & National Laboratory of Biomacromolecules, Institute of Biophysics, Chinese Academy of Sciences, Beijing 100101, China; 2 School of Life Sciences, Fujian Agriculture and Forestry University, Fuzhou 350002, China; 3 The Fourth People’s Hospital, Foshan 528000, China; 4 Shanghai Municipal Center for Disease Control and Prevention, Shanghai 200336, China; 5 State Key Laboratory of Virology, Wuhan Institute of Virology, Chinese Academy of Sciences, Wuhan 430071, China; 6 University of Chinese Academy of Sciences, Beijing 100049, China

## Abstract

DNA polymerase III (DNA pol III) is a multi-subunit replication machine responsible for the accurate and rapid replication of bacterial genomes, however, how it functions in *Mycobacterium tuberculosis* (*Mtb*) requires further investigation. We have reconstituted the leading-strand replication process of the *Mtb* DNA pol III holoenzyme *in vitro*, and investigated the physical and functional relationships between its key components. We verify the presence of an αβ_2_ε polymerase-clamp-exonuclease replicase complex by biochemical methods and protein-protein interaction assays *in vitro* and *in vivo* and confirm that, in addition to the polymerase activity of its α subunit, *Mtb* DNA pol III has two potential proofreading subunits; the α and ε subunits. During DNA replication, the presence of the β_2_ clamp strongly promotes the polymerization of the αβ_2_ε replicase and reduces its exonuclease activity. Our work provides a foundation for further research on the mechanism by which the replication machinery switches between replication and proofreading and provides an experimental platform for the selection of antimicrobials targeting DNA replication in *Mtb*.

In spite of extensive efforts, tuberculosis (TB), caused by the pathogen *Mycobacterium tuberculosis* (*Mtb*), remains a significant global public health threat^1^; in 2013, there were 9 million incident cases of TB and 1.5 million deaths^2^. The situation is further aggravated by the emergence of multidrug-resistant (MDR) and extensively drug resistant (XDR) TB, co-infection with HIV, and the low efficacy of the Bacille-Calmette-Guerin (BCG) vaccine^3^. There is an urgent need for new drugs. DNA polymerase III (DNA pol III), responsible for chromosomal DNA replication in all eubacteria including *Mtb*^4^, plays an important role in bacterial proliferation and is thus a significant and promising drug target; however, its mechanism in *Mtb* requires further investigation.

The DNA polymerase III system of *Mtb* is a large multisubunit machine, containing at least 6 subunits; α, ε, β, τ/γ, δ and δ′^5^. Genes encoding these subunits have been annotated in the *Mtb* genome, but the θ, χ and ψ subunits present in *E. coli* DNA Pol III, the most extensively studied model of DNA pol III, are absent in *Mtb* as in most other Gram-positive bacteria^4^,^6^. While only one α subunit (DnaE) is present in *E. coli* DNA pol III, two distinct homologues of the *E. coli* α subunit, DnaE1 and DnaE2, have been identified in the *Mtb* genome^7^. DnaE2 (also named ImuC) is a nonessential error-prone polymerase^8^,^9^, and DnaE1 is considered to be the DNA polymerase responsible for faithful genome replication. A 3-D structural model of *Mtb* α (MtbDnaE1) in complex with a small molecule inhibitor confirmed its structural differences from the human genomic replicase, and thus its promise as a drug target^10^. The crystal structure of the β_2_ clamp, the classical processive factor of DNA pol III, has been solved in *Mtb* at resolutions of 2.89 Å^11^ and 3.00 Å^12^, likewise confirming its close homology, including binding sites for α and other subunits, with the β_2_ clamp of *E. coli*. Little, however, is known about the structure and function of the other subunits of *Mtb* DNA pol III.

The functional efficiency of DNA pol III is determined by its replication rate, fidelity and processivity, characteristics that affect bacterial proliferation rates and the frequency of mutations in genes and intergenic regions which lead to drug-resistance^13^. The replicative fidelity of DNA pol III, determined by base selection by the α polymerase^14^ and editing of polymerase errors by proofreading factor(s)^15^, is of great importance in *Mtb* as it lacks a DNA mismatch repair (MMR) system^16^. Based on studies in *E. coli,* the proofreading activity of bacterial DNA Pol III has long been attributed to the ε exonuclease, a 3′–5′ exonuclease bound to the α subunit^17^, which increases its replication fidelity by about 10^2^–10^3^ fold^18^. However, accumulating evidence suggests that exonuclease activity residing in the PHP (polymerase and histidinol phosphatase) domain of the α subunit of many bacteria may actually be the ancestral prokaryotic proofreader^19^,^20^,^21^. Rock *et al.* recently reported that this αPHP domain exonuclease activity is responsible for proofreading during DNA replication in *Mtb* and appears to eliminate any role for mycobacterial DnaQ homologues under standard culture conditions *in vitro*^21^. However, which of these two exonuclease activities (ε and α) fulfills the proofreading role in *Mtb* DNA pol III *in vivo*, or whether both may be involved, requires further investigation. A delicate balance between the exonuclease activity of DNA pol III and its polymerase activity is necessary to maintain both its replicative rate and fidelity. In *E. coli* this balance is achieved as the αεθ:β_2_ replicase complex, formed when the αεθ core of DNA pol III associates with the β_2_ clamp, switches between polymerization and proofreading modes^22^,^23^,^24^ and the interactions between the α, ε and β_2_ subunits, especially the ε-β_2_ interaction, likely play an important role in this switch^22^,^23^. The physical and functional interactions between α, ε and β_2_ in *Mtb* DNA pol III and the mechanism by which *Mtb* DNA pol III regulates the balance between polymerase and exonuclease activity remain to be elucidated.

Here, in order to characterize *Mtb* DNA pol III, we reconstituted the leading-strand replication process of the *Mtb* DNA pol III holoenzyme *in vitro* and used standard protein-protein interaction assays and exonuclease and primer-extension assays to investigate the physical and functional relationships between its key components. We show that β_2_ may play an important bridging role between α and ε, both of which have ssDNA exonuclease activity and may serve as proofreading subunits. Our findings provide important insights into the mechanism by which *Mtb* DNA pol III transitions between polymerization and proofreading modes; the presence of the β_2_ clamp contributes to maintaining the αβ_2_ε replicase in polymerization mode and conditions required for ongoing polymerization (i.e. the presence of adequate amounts of dNTPs) may be essential for the transition from proofreading to polymerization mode.

## Results

### Reconstitution of leading-strand replication by Mtb DNA pol III holoenzyme

**Figure 1:**
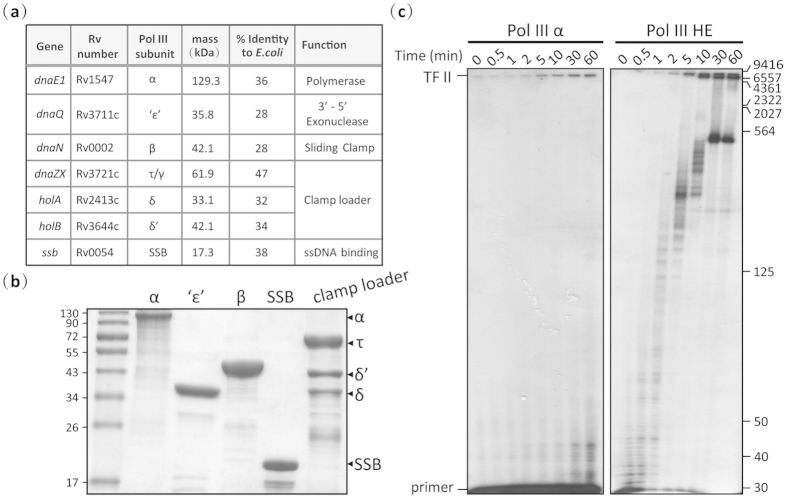
Purification of the protein subunits of the *Mtb* DNA pol III holoenzyme and reconstitution of leading-strand replication. (**a**) Gene name, mass and predicted function of *Mtb* DNA Pol III subunits, and their percent identity to corresponding subunits in the *E. coli* Pol III system^25^. (**b**) Coomassie Blue-stained 12% SDS-polyacrylamide gel of the purified DNA pol III holoenzyme subunits. The positions of subunits in the gel are indicated by arrows. All purified proteins were verified by peptide mass fingerprinting (PMF) using matrix-assisted laser desorption/ionization time-of-flight mass spectrometry (MALDI-TOF MS). **(c)** Reconstitution of leading-strand replication by the α subunit alone (left panel) and by the holoenzyme (right panel). The position of the fully extended M13mp18 DNA (TF II, 7249 bp) is indicated on the left. Size markers are shown on the right. Results presented are representative of at least three replicate experiments.

Genes corresponding to typical DNA Pol III subunits (α (*dnaE1*), ε (*dnaQ*), β (*dnaN*), τ (*dnaZX*), δ (*holA*), δ’ (*holB*), and SSB (*ssb*)) have been annotated in the *Mtb* genome^5^ (Fig. 1a)^25^; however, with the exception of the α^10^,^21^ and β subunits^11^, little functional information is available. In *E. coli*, the subunits of DNA pol III are organized into three functional parts: the polymerase core (αεθ), a ring-shaped sliding-clamp (β_2_) and a clamp loader (τ_2_γδδ’ ψχ)^4^. To maintain high efficiency, the main catalytic α subunit of *E. coli* DNA pol III has to interact with other subunits, such as ε^17^, β^24^, τ^26^ and SSB^27^, to form a holoenzyme. Here, we purified these *Mtb* DNA Pol III subunits (Supplementary Experimental Methods; Fig. 1b) and reconstituted the leading-strand replication activity of the *Mtb* holoenzyme. All subunits expressed well in *E. coli*, however, unlike the *dnaX* gene in *E. coli* which encodes two subunits, γ and τ, the *Mtb dnaZX* gene expressed in *E. coli* only produced one protein, the τ subunit. In addition, cells expressing τ, δ, and δ’ had to be co-lysed in order to purify a stable clamp loader complex. Densitometric scanning of an SDS-PAGE gel indicated that τ, δ and δ’ were present in a 3:1:1 ratio in the clamp loader complex, as in *E. coli*^28^. To further verify the integrity and activity of our *Mtb* DNA pol III holoenzyme, we reconstituted leading-strand replication using a M13mp18 ssDNA template (7249 bp). While *Mtb* α on its own could catalyze the DNA extension reaction (Fig. 1c, left panel), confirming that it is the main catalytic subunit, a highly efficient DNA polymerase with high rate and processivity was only reconstituted in the presence of the other DNA pol III components (Fig. 1c, right panel).

### *Mtb* DNA Pol III ‘ε’ is a Mg^2+^-dependent exonuclease with a preference for ssDNA

**Figure 2:**
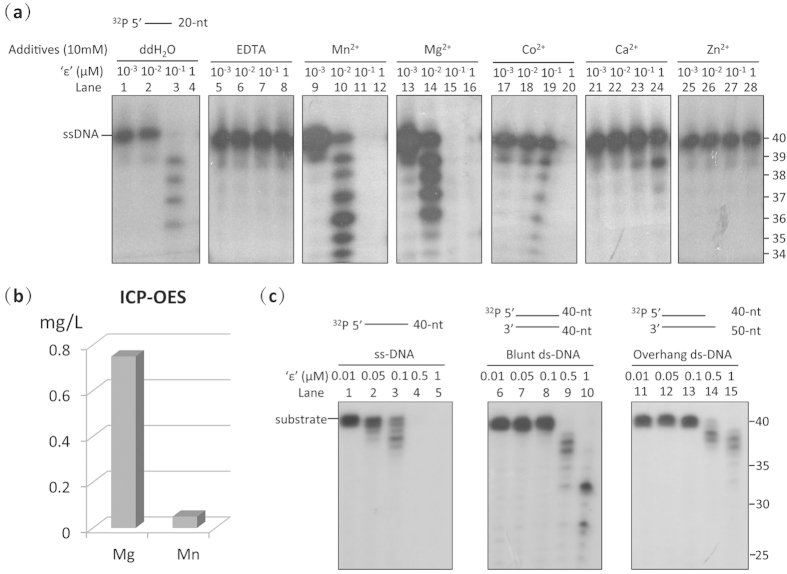
The ‘ε’ subunit of *Mtb* DNA pol III has exonuclease activity. (**a**) Ion dependency of the exonuclease activity of the ‘ε’ subunit. The ‘ε’ subunit is a 3′–5′ exonuclease which is activated by Mg^2+^ and Mn^2+^, and inhibited by EDTA, Co^2+^, Ca^2+^and Zn^2+^. (**b**) ICP-OES quantitation of metal ions in the purified ‘ε’ subunit. The native ‘ε’ subunit binds significantly more Mg^2+^ (~0.75 mg/L) than Mn^2+^ (less than 0.05 mg/L), and is thus a Mg^2+^-dependent exonuclease. The mean of three replicate samples analysed is shown. (**c**) The exonuclease activity of the ‘ε’ subunit shows a preference for ssDNA over blunt-dsDNA or 5′ overhanging-dsDNA as substrate. Substrate types are shown above each panel. Results presented are representative of at least three replicate experiments.

We next examined whether Rv3711, the most likely *Mtb* homologue of the ε proofreading subunit of *E. coli* DNA pol III (hereafter referred to as ‘ε’) has exonucleolytic activity as recently reported^21^. Sequence alignment of Rv3711 with ε homologues from other bacteria indicated that it has a highly conserved exonuclease active site containing six residues (D20, E22, D104, R155, H158 and D163) in its N-terminal domain (Supplementary Fig. S1). Assaying the exonuclease activity of ‘ε’ using 5′-labelled single-stranded DNA indicated that, similar to the *E. coli* ε exonuclease^29^, ‘ε’ has 3′–5′ DNA exonuclease activity that is inhibited by EDTA (Fig. 2a, Lanes 1–8), indicating that its activity is likely divalent metal ion-dependent. Subsequent experiments indicated that ‘ε’ is strongly activated in the presence of Mn^2+^ and Mg^2+^, and its exonuclease activity is slightly higher in the presence of Mn^2+^ than Mg^2+^ (Fig. 2a, Lanes 9–16), in agreement with previous studies on *E. coli* ε^29^,^30^, while its activity decreased to different degrees in the presence of Co^2+^, Ca^2+^ and Zn^2+^ (Fig. 2a, Lanes 17–28). To further clarify the importance of Mn^2+^ and Mg^2+^ in the exonuclease activity of native ‘ε’, we measured the quantities of these ions in ‘ε’ using ICP-OES. The quantity of Mg^2+^ was about one order of magnitude greater than that of Mn^2+^ in native ‘ε’ (Fig. 2b), indicating that the metal ions in the active site of *Mtb* ‘ε’, as in *E.coli* ε^30^, are Mg^2+^ ions rather than Mn^2+^ ions.

When we examined the substrate preference of ‘ε’, results indicated that ‘ε’ excises the 3′-terminus of single-stranded DNA considerably faster (Fig. 2c, Lanes 1–5) than that of paired dsDNA, irrespective of whether the dsDNA had a blunt end or 5′-overhang (Fig. 2c, Lanes 6–15). The *Mtb* ‘ε’ exonuclease thus has a preference for ssDNA, and is probably involved in proofreading.

### *Mtb* DNA Pol III α has exonuclease as well as polymerase activity

**Figure 3:**
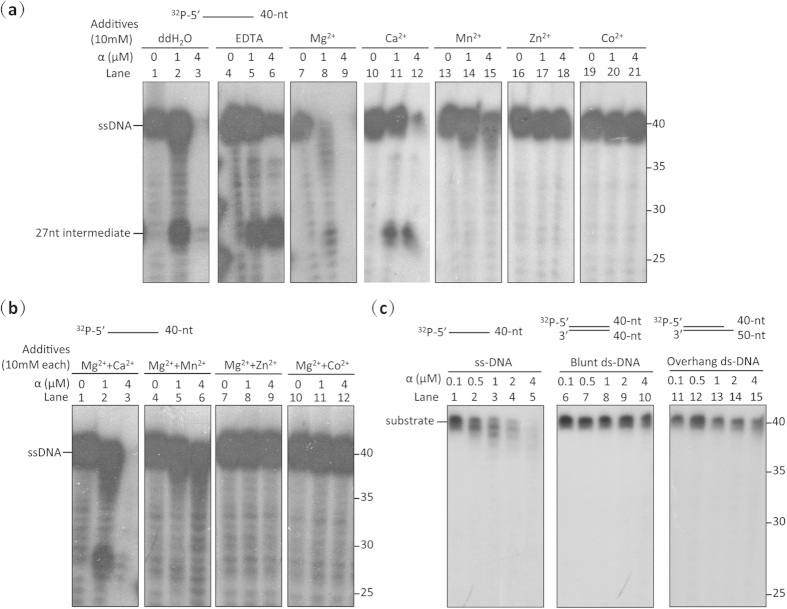
The α subunit of *Mtb* DNA Pol III has exonuclease activity. (**a**) Exonuclease assays indicate that the α subunit is a 3′–5′ exonuclease which is activated by Mg^2+^, and inhibited by EDTA, Ca^2+^, Mn^2+^, Zn^2+^ and Co^2+^. (**b**) Assay of the exonuclease activity of the α subunit in the presence of Mg^2+^ combined with other divalent metal ions. The presence of Mg^2+^ does not affect the sensitivity of the exonuclease activity of the α subunit to Ca^2+^, Mn^2+^, Zn^2+^ or Co^2+^. (**c**) The DNA Pol III α subunit is a single-stranded DNA exonuclease as determined by assays using ssDNA, blunt-dsDNA or 5′ overhanging-dsDNA as substrates. Substrate types are shown above each panel. Results presented are representative of at least three replicate experiments.

Accumulating evidence also points to the presence of a highly conserved exonuclease active site in the PHP domain of the α subunit of bacterial DNA pol III^19^,^20^,^21^. Initial evidence from a study on the α subunit of *T. thermophilus* DNA pol III which showed it to have an intact exonuclease active site in its PHP domain and Zn^2+^-dependent exonuclease activity, suggested that it may be the proofreading subunit of DNA pol III^19^. A recent phylogenetic analysis of α and ε homologues across the bacterial kingdom indicated that the majority of replicative bacterial polymerases contain an active PHP exonuclease site in the α subunit while *E. coli*-like ε exonucleases appear to be unique to the *α-*, *β-* and *γ-*proteobacteria^21^. Sequence alignment of the *Mtb* α subunit with that of other bacteria here (Supplementary Fig. S2) confirmed the presence of an intact PHP exonuclease active site consisting of nine residues (H14, H16, D23, H48, E73, H107, C158, D226 and H228), and exonuclease activity assays using 5′-labelled single-stranded DNA verified that it has 3′–5′ exonuclease activity (Fig. 3a, Lanes 1–3), confirming that, in addition to its polymerase activity, *Mtb* α is likely also involved in proofreading^21^. In contrast to ‘ε’, the exonuclease activity of α was not absolutely inhibited by a large excess of EDTA (Fig. 3a, Lanes 4–6). It was, however, strongly activated by the addition of Mg^2+^ (Fig. 3a, Lane 7–9), indicating that it is a Mg^2+^-activated 3′–5′ DNA exonuclease. The observed exonuclease activity was inhibited completely by Zn^2+^ (Fig. 3a, Lanes 16–18), and to lesser degrees by the other metal ions tested (Ca^2+^, Mn^2+^, Zn^2+^ and Co^2+^) (Fig. 3a, Lanes 10–21). The catalytic divalent ions residing in the PHP domain of *Mtb* α (pol III) are thus most likely to be Mg^2+^ rather than Zn^2+^ as is the case for α (pol III) in *T. thermophilus*^19^ or Mn^2+^ as is the case in the Pol X of *T. thermophilus*^31^ and *B. subtilus*^32^. These results eliminate any possibility that the *Mtb* α subunit used here became contaminated with *E. coli* ε subunits during protein purification; EDTA only partially inhibited the exonuclease activity of the *Mtb* α subunit (Fig. 3a, Lanes 4–6) while it is known to completely inhibit the exonuclease activity of *E. coli* ε^29^, and while *E. coli* ε is known to be activated by Mn^2+^ 30, the exonuclease activity of *Mtb* α was considerably inhibited by Mn^2+^ (Fig. 3a, Lanes 13–15). In addition, cleavage of DNA by the α exonuclease showed a temporary pause at ~27 nt (Fig. 3a, Lane 2), coincident with the length of DNA occupied by the α subunit^20^, further demonstrating that the exonuclease activity arose from *Mtb* α. To rule out the possibility that the reduction in exonuclease activity of *Mtb* α observed here in the presence of each ion tested (except Mg^2+^) resulted from conformational change due to coordination of insufficient Mg^2+^ ions in the Palm polymerase active site, we measured its exonuclease activity in the presence of the tested ions (Ca^2+^, Mn^2+^, Zn^2+^ and Co^2+^) in combination with Mg^2+^. Sensitivity of the exonuclease activity to the tested ions was barely affected by the presence or absence of Mg^2+^ (Fig. 3a, Lanes 10–21; and Fig. 3b), indicating that α exonuclease activity is directly inhibited by the four ions themselves.

We next examined the substrate preference of the α exonuclease. Results indicated that α preferentially hydrolyses ssDNA rather than matched duplex DNA (Fig. 3c), further supporting its proposed role for removing mispaired-nucleotides. Since degradation of all radioactive ssDNA substrates was accomplished here at much lower concentrations of ‘ε’ (Fig. 2c, Lane 4) than of α (Fig. 3c, Lane 5), the exonuclease activity of *Mtb* ‘ε’ appears to be higher under the experimental conditions used here than that of α. Taken together, then, these findings indicate that *Mtb* DNA Pol III α and ‘ε’ may both fulfill proofreading roles.

### The α polymerase, ‘ε’ exonuclease and β clamp physically interact with each other in *Mtb* to form an αβ_2_ ‘ε’ replicase

**Figure 4:**
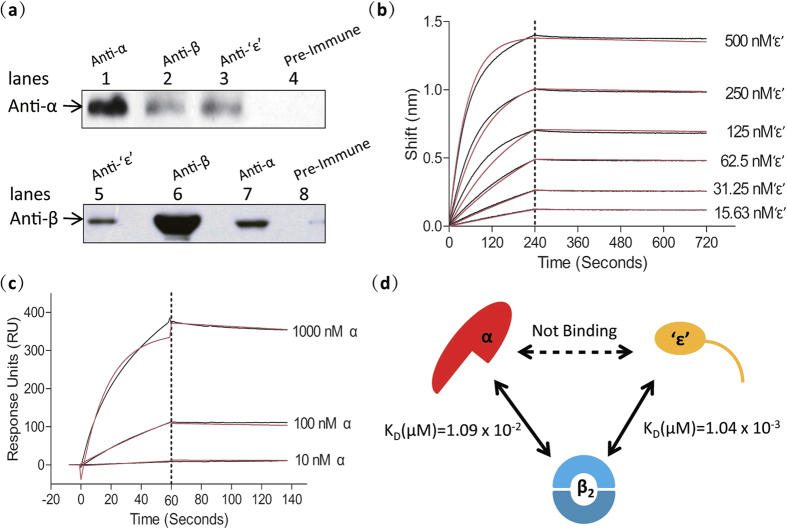
The subunits of the αβ_2_‘ε’ complex of *Mtb* DNA pol III interact physically both *in vitro* and *in vivo*. (**a**) Physical interactions between α, β_2_ and ‘ε’ as determined by Co-IP. Sera incubated with cell lysate supernatants are indicated above the panels. Sera used as primary antibodies in Western blotting are indicated on the left. Lanes 4 and 8 are negative controls. Lanes 1 and 6 are positive controls and confirm the specificity of the anti-α and anti-β sera. (**b**) Kinetics of the ‘ε’-β interaction (K_D_ = 1.04 × 10^−3^ μM) as determined by BLI using six concentrations of the ‘ε’ subunit (15.63, 31.25, 62.5, 125, 250 and 500 nM). Black lines represent the data before fitting, and red lines represent the global fit of the entire data set to a 1:1 Langmuir interaction model. (**c**) Kinetics of the α-β interaction (K_D_ = 1.09 × 10^−2^ μM) as determined by SPR using three concentrations of the α subunit (0.01, 0.1 and 1 μM). Black lines represent the data before fitting, and red lines represent the global fit of the entire data set to a 1:1 Langmuir interaction model. (**d**) Diagram showing the protein interactions between the subunits of the αβ_2_‘ε’ complex and their kinetic parameters determined *in vitro*. While the α subunit does not bind to the ‘ε’ exonuclease subunit, they are both tightly bound to the β_2_ clamp. Results presented are representative of at least three replicate experiments.

As the presence of a proofreading ε subunit in *E. coli* and other *α-*, *β-* and *γ-*proteobacteria is correlated with a defective PHP domain in the α subunit, there is debate as to whether an ε exonuclease and αPHP exonuclease can co-exist^19^,^21^,^33^. However, a recent bioinformatic analysis reported the presence of an intact PHP active site and a conserved putative ε-binding patch on the surface of the PHP domain in the DNA pol III of bacteria such as the *Firmicutes*, supporting the possibility of co-existence^7^. To determine whether the exonuclease activity of the α and ‘ε’ subunits is mutually exclusive in *Mtb* we first investigated the physical interactions between α, ‘ε’ and β_2_ *in vivo* by co-immunoprecipitation (Co-IP) and *in vitro* by Biolayer interference (BLI), Surface plasmon resonance (SPR) and Isothermal Titration Calorimetry (ITC). Co-IP confirmed that α, ‘ε’ and β_2_ interact with each other, likely forming an αβ_2_ ‘ε’ ternary complex *in vivo* (Fig. 4a) similar to the αεθ:β_2_ replicase of *E. coli*^34^. BLI and ITC results, however, indicated that ‘ε’ does not interact with α, (Supplementary Fig. S3), in contrast to the situation in *E. coli* where stable α-ε complexes have been purified^17^, but in agreement with recent findings by Rock *et al.*^21^. To investigate why the α-ε interaction disappears in *Mtb*, we aligned the sequences of these subunits with those of their homologues in other bacteria. Sequence alignments suggest that while the hydrogen bond between ε-H255 and α-K63 involved in the α-ε interaction in *E. coli*^7^ is conserved in *Mtb*, a tryptophan in *Mtb* ‘ε’ in an analogous position to the ε-Trp241 in *E. coli* which binds to the αPHP domain is absent, providing a possible explanation for the loss of the α-‘ε’ interaction (Supplementary Fig. S1). We reason that the conflicting results obtained here using Co-IP and BLI may be explained by the existence of a bridging protein that tightly couples α and ‘ε’. BLI results indicate that *Mtb* ‘ε’ binds tightly to β_2_ with a K_D_ of 1.04 × 10^−3^ μM, an interaction that is five orders of magnitude stronger than that found in *E. coli* (K_D_ = ~200 μM)^22^ (Fig. 4b). To identify the residues of ‘ε’ responsible for the interaction with β_2_, we analyzed the ‘ε’ sequence using PSIPRED and DISOPRE; the C-terminal segment of ‘ε’ is predicted to be disordered and capable of binding proteins (Supplementary Fig. S4). Likewise, *Mtb* α binds to β_2_ with a K_D_ (1.09 × 10^−2^ μM) that is much stronger than that reported for the binding of α to β_2_ in *E. coli* (K_D_ = 1.2 ± 0.2 μM)^35^,^36^ (Fig. 4c). These results thus suggest that β_2_ is associated with both α and ‘ε’, and likely plays an important bridging role in the formation of an αβ_2_‘ε’ replicase in *Mtb* (Fig. 4d). These interactions between α, ‘ε’ and β_2_ also imply that the exonuclease activity of ‘ε’ and the PHP domain of α are not exclusive, further confirming that they are both potential proofreading factors in *Mtb* DNA Pol III.

### Equimolar mixtures of *Mtb* DNA Pol III α and ‘ε’ exhibit unexpected exonuclease activity on paired duplex DNA *in vitro*

**Figure 5:**
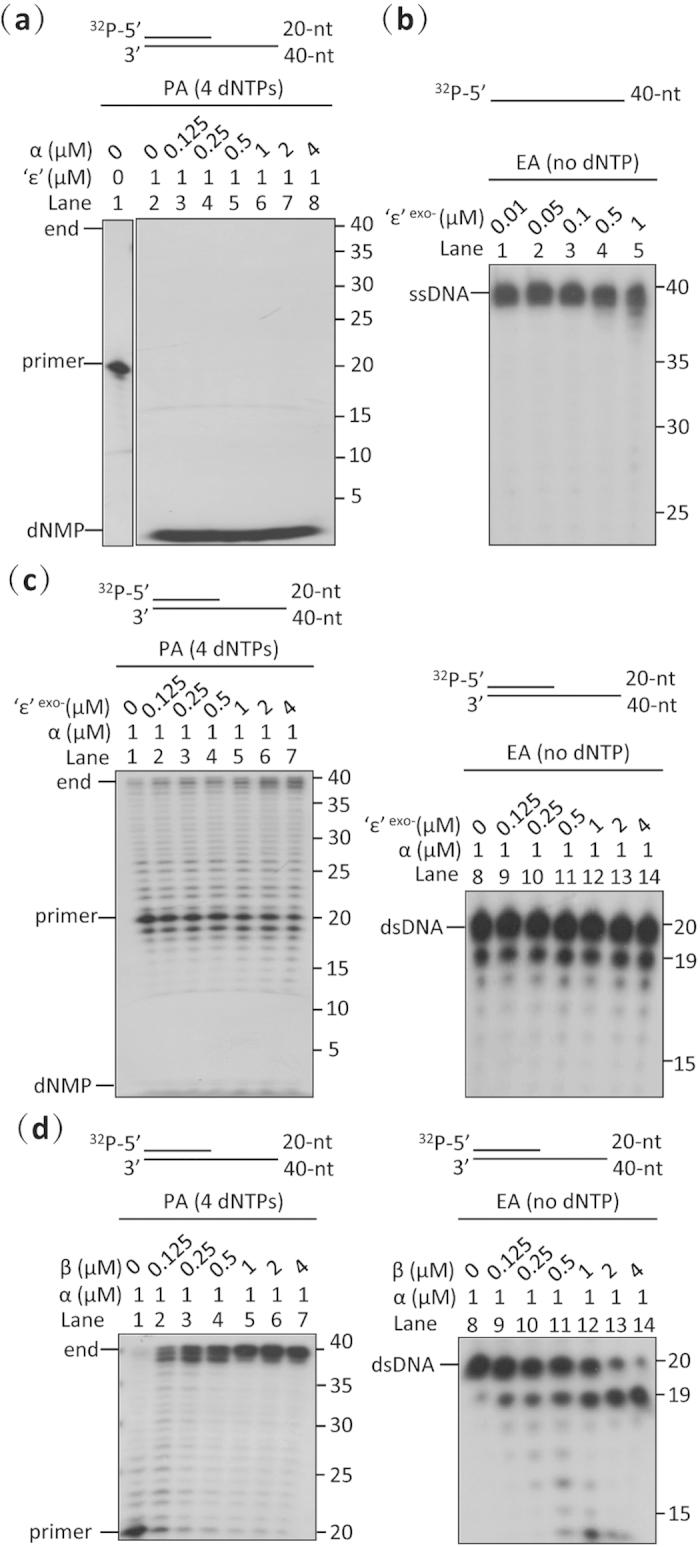
Investigation of the functional interactions between the subunits of the αβ_2_‘ε’ complex of *Mtb* DNA pol III. (**a**) Primer-extension assay of different ratios of α and ‘ε’ subunits using a primed dsDNA substrate. Mixtures of α and ‘ε’ subunits exhibit unexpectedly high exonuclease activity on paired duplex DNA and no polymerase activity *in vitro*. Increasing amounts of the α subunit fail to weaken the exonuclease activity of the ‘ε’ subunit *in vitro*. (**b**) Exonuclease assay of the ‘ε’ mutant (‘ε’^exo-^) using a ssDNA substrate. Mutation of the active site of the ‘ε’ subunit (D20A, E22A and D104A) successfully destroys its exonuclease activity. (**c**) Primer-extension and exonuclease assays of the α subunit with increasing amounts of ‘ε’^-exo^ using a primed dsDNA substrate. The ‘ε’^-exo^ mutant modestly enhances the polymerase activity of the α subunit (left panel), but has no effect on its exonuclease activity (right panel). (**d**) Primer-extension and exonuclease assays of the α subunit with increasing amounts of β_2_ clamp using a primed dsDNA substrate. While the polymerase activity of the α subunit is strongly promoted by the presence of the β_2_ clamp (left panel), its exonuclease activity is only slightly increased (right panel). The reaction conditions of the polymerase (PA) and exonuclease assays (EA) were the same, except that the 4 dNTPs were omitted in the exonuclease assay. The primed dsDNA substrate was produced by annealing a 5’-^32^P-labeled ssDNA primer (20-mer) to an unlabeled ssDNA template (40-mer). Results presented are representative of at least three replicate experiments.

We next investigated whether, as in *E. coli*, *Mtb* α and ‘ε’ together might execute both polymerase and exonuclease activities. While the reconstituted *E. coli* αε core chiefly exhibits polymerase activity^37^, an equimolar mixture of *Mtb* α and ‘ε’ showed unexpectedly strong exonuclease activity on paired dsDNA and no polymerase activity (Fig. 5a, Lane 6). No primer extension was observed even when the molar ratio of α and ‘ε’ was increased to 4:1 (Fig. 5a, Lane 8). To verify which subunit was responsible for this unexpectedly strong exonuclease activity, we replaced the D20, E22 and D104 residues in the active site of ‘ε’ with alanine to destroy its exonuclease activity (Fig. 5b). When wild-type ‘ε’ (‘ε’^WT^) in the mixture was replaced by this ‘ε’^exo-^ mutant, the equimolar α-‘ε’^exo-^ mixture extended the primed DNA substrate and failed to fully degrade paired dsDNA into dNMP, even though it could still slowly remove nucleotides from the 3′ terminus (Fig. 5c, Lane 5). This indicates that the strong exonuclease activity of the α-‘ε’^WT^ mixture likely arises from ‘ε’ rather than α and that the weak exonuclease activity of the α-‘ε’^exo-^ mixture is likely catalyzed by α.

To understand the influence of ‘ε’ on the function of α, we examined the polymerase and exonuclease activities of α in the presence of increasing amounts of ‘ε’^exo-^. The presence of ‘ε’ (ε^exo-^) considerably enhanced the polymerase activity of α (Fig. 5c, left panel), in agreement with results from *E. coli*^37^,^38^. In contrast, the exonuclease activity of α was not affected by the addition of ‘ε’^exo-^ at concentrations of 0.125 μM to 4 μM (Fig. 5c, right panel), suggesting that the exonuclease activity of α, either alone or when together with ‘ε’, is not as strong as that of the equimolar α-‘ε’^WT^ mixture.

Given the tight physical interaction between α and β_2_, we investigated whether β_2_ has any direct influence on the exonuclease or polymerase activity of α. Increasing the concentration of β_2_ markedly increased the polymerase activity of α (Fig. 5d, left panel), mimicking the effect seen in *E. coli*^39^,^40^. On the other hand, the exonuclease activity of α increased only slightly in the presence of β_2_ (Fig. 5d, right panel). In addition, the α exonuclease only removed one nucleotide from the 3′ end of the DNA (Fig. 5d, right panel) and, unlike the ‘ε’ exonuclease, failed to further digest the DNA into dNMPs (Fig. 5a).

Taken together, these results indicate that, in addition to the observed physical ‘ε’-β_2_ and α-β interactions, ‘ε’ and β_2_ also interact functionally with α, enhancing its polymerase activity, with β_2_ promoting its polymerase activity to a greater extent than ‘ε’.

### β_2_ simultaneously promotes the polymerase and reduces the exonuclease activity of the αβ_2_‘ε’ replicase

Although *Mtb* ‘ε’ exhibits a distinct preference for mispaired DNA over paired DNA, as shown above, an equimolar α-‘ε’^WT^ mixture was still capable of rapidly degrading paired DNA (Fig. 5a, Lane 6). We thus hypothesized that there must be an underlying mechanism by which the exonuclease activity of ‘ε’ is modulated when it is in association with other subunits in a DNA Pol III subassembly or holoenzyme in the active replication fork.

**Figure 6:**
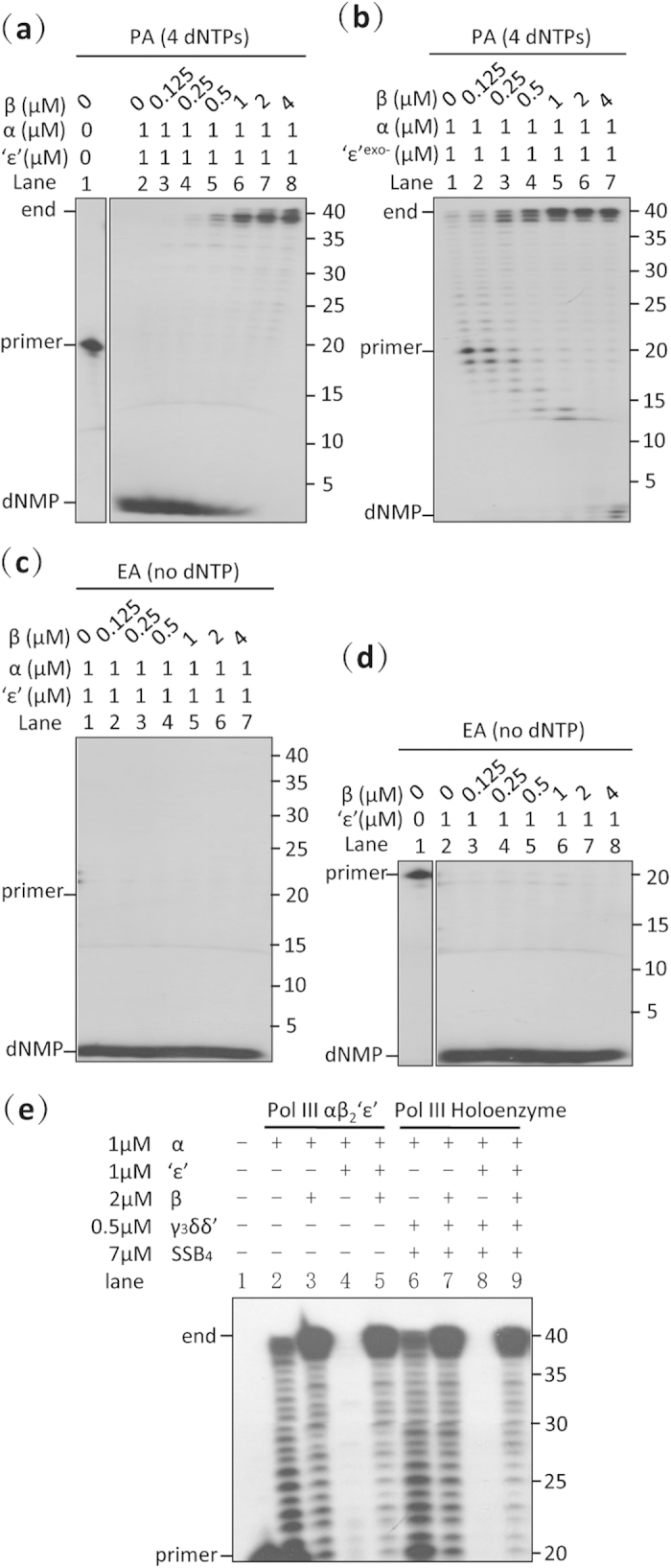
Role of the β_2_ clamp in the αβ_2_‘ε’ complex of *Mtb* DNA pol III. (**a**) Primer-extension assay of the αβ_2_‘ε’ replicase. The β_2_ clamp strongly promotes the polymerization and reduces the exonuclease activity of the αβ_2_‘ε’ complex. (**b**) Primer-extension assay of the αβ_2_‘ε’^exo-^ replicase. Once the exonuclease activity of the ‘ε’ subunit is mutated, the β_2_ clamp promotes the polymerase activity, and very slightly enhances the exonuclease activity of the αβ_2_‘ε’^exo-^ replicase. (**c**) Exonuclease assays of the αβ_2_‘ε’ replicase. Increasing the amount of the β_2_ clamp does not reduce the exonuclease activity of the αβ_2_‘ε’ complex in the absence of the 4 dNTPs. (**d**) Exonuclease assays of different ratios of ‘ε’ and the β_2_ clamp. Increasing the amount of the β_2_ clamp fails to directly preclude the exonuclease activity of the ‘ε’ subunit *in vitro*. (**e**) Primer-extension assay of *Mtb* DNA pol III subassemblies. Equimolar mixtures of the α and ‘ε’ subunits exhibited only exonuclease activity, both in the presence (holoenzyme) and absence (αβ_2_‘ε’) of the clamp loader and SSB, and the presence of the β_2_ clamp reduced exonuclease activity and restored polymerase activity. The reaction conditions of the polymerase (PA) and exonuclease assays (EA) were the same, except that the 4 dNTPs were omitted in the exonuclease assay. The primed dsDNA substrate used in all the above experiments was produced by annealing a 5′-^32^P-labeled ssDNA primer (20-mer) to an unlabeled ssDNA template (40-mer). Results presented are representative of at least three replicate experiments.

To determine whether the β_2_ clamp might play a role in regulating the polymerase/exonuclease activity of DNA polymerase III in addition to its bridging role in physically integrating α and ‘ε’ to stabilize the αβ_2_‘ε’ replicase, we examined the effect of gradually increasing the amount of β_2_ on the exonuclease and polymerase activity of an α-‘ε’^WT^ mixture. Gradually increasing the amount of β_2_ enhanced the polymerase activity of the α-‘ε’^WT^ mixture and reduced its exonuclease activity (Fig. 6a), indicating that the formation of the αβ_2_‘ε’ replicase complex leads to increased polymerase over exonuclease activity, and suggesting that the interaction of α and ‘ε’ with β_2_ may be indispensable for guaranteeing rapid DNA replication.

To determine if the enhanced polymerase activity of the αβ_2_‘ε’ replicase is directly linked to decreased exonuclease activity, we repeated the above experiment using the ‘ε’^exo-^ mutant. The polymerase activity of the α-‘ε’^exo-^ mixture was stimulated in the presence of β_2_ as was the case for the α-‘ε’^WT^ mixture (Fig. 6b), and the exonuclease activity of the α-‘ε’^exo-^ mixture also increased slightly (Fig. 6b). These findings indicate that promotion of the α-‘ε’^WT^ mixture polymerase activity by β_2_ likely does not rely directly on a decline in the exonuclease activity of the α-‘ε’^WT^ mixture. In addition, they suggest that the gradual enhancement of DNA synthesis observed with increasing amounts of β_2_ is due to the direct stimulation of the polymerase activity of α by β_2_ (Fig. 5d left panel) discussed above. The decline in exonuclease activity of the α-‘ε’^WT^ mixture in the presence of β_2_, then, is likely due to the β_2_ clamp directly or indirectly blocking the exonuclease activity of ‘ε’ rather than α; the reduced exonuclease activity of the α-‘ε’^exo-^ mixture failed to fully degrade the dsDNA substrate into dNMP, even in the presence of β_2_ (Fig. 6b). We further investigated the effect of the β_2_ clamp on ‘ε’ by examining the effect of increasing amounts of β_2_ on ‘ε’ in the absence of α. Results indicate that β_2_ does not directly inhibit the exonuclease activity of ‘ε’ in the absence of α (Fig. 6d).

Next, to investigate if there is a direct relationship between the decrease in the exonuclease activity of the α-‘ε’^WT^ mixture and the increase in its polymerase activity on addition of the β_2_ clamp, we blocked polymerase activity by omitting the four dNTPs. Interestingly, when all four dNTPs were removed, increasing the amount of β_2_ did not reduce the exonuclease activity of the α-‘ε’^WT^ mixture (Fig. 6c). To rule out the possibility that the dNTPs might somehow contribute to the decrease in exonuclease activity, we repeated the experiment using dGTP to replace the four dNTPs; the presence of β_2_ still failed to reduce the exonuclease activity of the α-‘ε’^WT^ mixture (data not shown). The decrease in exonuclease activity of the α-‘ε’^WT^ mixture in the presence of β_2_ thus appears to be directly linked to active ongoing DNA polymerization catalyzed by α.

To determine whether the direct regulatory effect of β_2_ on polymerization and exonuclease activity occurs in both the αβ_2_‘ε’ complex and the holoenzyme of the *Mtb* DNA pol III, we examined the polymerase and exonuclease activity of the αβ_2_‘ε’ complex in the presence of SSB and the β_2_ clamp loader. Exonuclease activity of the α-‘ε’ mixture was reduced in the presence of the β_2_ clamp and its polymerase activity was restored in both the αβ_2_‘ε’ complex and the holoenzyme of the *Mtb* DNA pol III, indicating that the formation of the DNA Pol III holoenzyme does not influence the role of β_2_ in regulating the polymerase and exonuclease activity of α and ‘ε’ (Fig. 6e).

## Discussion

The *Mtb* DNA polymerase III system, responsible for the accurate and rapid replication of its chromosomal DNA and thus a significant and promising drug target, is poorly understood. Here, we have successfully reconstituted leading-strand replication of the *Mtb* DNA pol III holoenzyme *in vitro*, and systematically characterized the physical and functional relationships between α, ‘ε’ and β_2_, its key components. We demonstrate that ‘ε’, like its *E. coli* counterpart^30^, is an exonuclease (Fig. 2a) and confirm recent findings that α has 3′–5′ ssDNA exonuclease activity in addition to its 5′–3′ DNA polymerase activity^21^ (Fig. 3a). We show that α becomes a highly efficient DNA polymerase only when associated with ‘ε’, the sliding clamp and clamp loader to form the holoenzyme (Fig. 1c). The β_2_ clamp of *Mtb* DNA pol III interacts tightly with both α and ‘ε’ (Fig. 4), and its presence appears to play an important regulatory role in the function of the αβ_2_‘ε’ replicase, increasing its polymerase and reducing its exonuclease activity. Our findings provide novel insights into the mechanism by which these two activities are balanced in *Mtb* DNA pol III, and open up new avenues of research on the proliferation and emergence of drug-resistance in this pathogen.

Our study provides direct experimental evidence that a bacterial DNA pol III can have two potential proofreading subunits. The ssDNA exonuclease activity of Rv3711 (‘ε’) (Fig. 2a), its strong physical interaction with the β_2_ clamp (Fig. 4b), the fact that ‘ε’ considerably enhances the polymerase activity of the α subunit (Fig. 5c), and that its own exonuclease activity is reduced by β_2_ in the presence of α (Fig. 6a) lead us to conclude that Rv3711 (‘ε’) is the *Mtb* homologue of the *E. coli* ε subunit and an integral part of a core αβ_2_ε replicase in *Mtb*. The presence of exonuclease activity in both α and ‘ε’ in *Mtb* DNA pol III (Figs. 2 and 3), then, is different from the sole ε proofreading subunit of *E. coli* and the sole αPHP proofreading subunit in *T. thermophilus*^31^. The presence of an exonuclease active site in the αPHP domain, as in the *T. thermophilus* α subunit, has previously been thought to preclude the involvement of the ε exonuclease in DNA pol III^33^. Here, we verified that the *Mtb* DNA pol III α subunit, like the *T. thermophilus* α subunit^19^, and in agreement with the findings of Rock *et al.*^21^, has an intact PHP active site which also has 3′–5′ ssDNA exonuclease activity (Fig. 3a). Our findings show that exonuclease activity in *Mtb* DNA pol III ‘ε’ and α is not mutually exclusive and both probably serve as proofreading subunits in genomic replication. While the exonuclease activity of ‘ε’ here was much stronger than that of α in *in vitro* assays (Figs. 2c and 3c), Rock *et al*’s recent work published during the preparation of this paper suggests that α may actually execute the main proofreading activity in living cells and may be an ancestral prokaryotic proofreader^21^.

In contrast to *E. coli* where α, ε and β_2_ all physically interact with each other *in vitro*, we found that *Mtb* α does not directly associate with ‘ε’ (Supplementary Figure S3), the β_2_ clamp playing an important bridging role in the αβ_2_‘ε’ replicase connecting α and ‘ε’ (Fig. 4). In *E. coli*, the α-ε interaction is strong enough to support a stable αε complex^17^, and plays an important role in the formation of the αεθ:β_2_ replicase^34^. Here, co-immunoprecipitation assays demonstrated that α, ‘ε’ and β_2_ form an αβ_2_‘ε’ replicase *in vivo* (Fig. 4a), similar to the αεθ:β_2_ replicase of *E. coli*. α-β_2_ and ‘ε’-β_2_ interactions are much stronger in *Mtb* than those in *E. coli*^22^,^36^ and may be essential for the formation of the *Mtb* αβ_2_‘ε’ replicase (Fig. 4b,c). The organization of the *Mtb* αβ_2_‘ε’ replicase reported here provides a physical foundation for elucidating the dynamic assembly of the *Mtb* DNA pol III subunits during the functional switches between replication and proofreading.

As in *E. coli*, functional interactions in the αβ_2_‘ε’ replicase of *Mtb* DNA pol III are important; ‘ε’ and β_2_ are necessary for α to maintain its native replication rate (Fig. 5c,d). In *E. coli* DNA pol III, the association of α and ε increases α polymerase activity by about 3-fold, and the ε exonuclease by 10- to 80-fold^37^, and the association of the β_2_ clamp with α also increases its polymerase activity^40^. Here, as in *E. coli*, both β_2_ and ‘ε’ are able to increase the polymerase activity of α, with β_2_ promoting the polymerase activity much more strongly than ‘ε’ (Fig. 5c,d). We also found that β_2_ is unable to reduce ‘ε’ exonuclease directly (Fig. 6d). Although we were unable to determine if α enhances the exonuclease activity of ‘ε’ in *Mtb* as it does in *E. coli*, we have shown that α does at least not reduce its activity (Fig. 5a).

Our results suggest that *Mtb* DNA pol III may be a good model for studying the mechanism by which DNA pol III switches from proofreading to polymerization mode; an equimolar mixture of *Mtb* DNA pol III α and ‘ε’ natively exhibits strong exonuclease activity on paired dsDNA (Fig. 5a, Lane 6), whereas the αε complex of *E. coli* DNA pol III mainly exhibits polymerase activity and has extremely weak exonuclease activity on paired dsDNA^37^. In *E. coli*, DNA pol III regulates these two activities by switching between polymerization and proofreading modes^22^,^23^. Jergic *et al.* have proposed that interactions between the α, ε and β_2_ subunits, especially an intact ε-β_2_ interaction, are essential for stabilizing the replicase in polymerization mode, and that disruption of the ε-β_2_ interaction in the *E. coli* αεθ:β_2_ replicase is necessary for the switch from polymerization mode to proofreading mode when sufficient amounts of the four dNTPs are present in the reaction^22^. However, Toste Rêgo *et al.* have reported that an intact ε-β interaction is also required for DNA pol III to maintain optimal proofreading activity when the polymerase runs out of dNTPs, and suggest that an intact ε-β_2_ interaction may play an important role in stabilizing the replicase in proofreading mode by positioning the ε exonuclease closer to the DNA substrate^23^. Our results suggest that the presence of the β_2_ clamp may play an important regulatory role in balancing the polymerase and exonuclease activities of DNA pol III in *Mtb*; the presence of the β_2_ clamp in the *Mtb* αβ_2_‘ε’ replicase promoted polymerase activity and reduced exonuclease activity (Fig. 6a), suggesting that β_2_ facilitates the switch in *Mtb* DNA pol III from proofreading to polymerization mode. This promotion of polymerase activity is likely due to a direct enhancing effect of the β_2_ clamp on the α polymerase in the absence of ‘ε’ (Fig. 5d). The β_2_ clamp’s reduction of exonuclease activity likely requires ongoing DNA polymerization as it does not reduce ε exonuclease activity in the absence of α or the four dNTPs (Fig. 6c,d). This observed regulatory role of β_2_ on the polymerase and exonuclease activities of *Mtb* DNA pol III, especially its reduction of exonuclease activity, may be important for *Mtb* DNA pol III to replicate the genome rapidly (Figs 5a and 6a).

In addition, we found that whether conditions are suitable or not for ongoing polymerization (the presence of enough dNTPs) may also affect the regulation of the polymerase and exonuclease activities of *Mtb* DNA pol III. We found that when conditions were suitable for DNA polymerization (when the four dNTPs were added), the presence of β_2_ contributed to the transition of DNA pol III from proofreading to polymerization mode (Fig. 6a), in agreement with the observations of Jergic *et al.* in *E. coli*^22^. However, when conditions were unsuitable (the four dNTPs were absent), the exonuclease activity of *Mtb* DNA pol III was not reduced by the presence of β_2_ (Fig. 6c), an observation which does not contradict Toste Rêgo *et al.*^23^. The ‘ε’-β interaction may also play an important role in this process as suggested by Toste Rêgo *et al.*^23^, but further study is required to confirm this. We conclude that, in addition to the interactions between subunits, conditions suitable for ongoing polymerization may be required for *Mtb* DNA pol III to switch from proofreading to polymerase mode, and speculate that the differences observed by Jergic *et al.* and Toste Rêgo *et al.* in the effects of the ε-β interaction on regulating the polymerase and exonuclease activities of *E. coli* DNA pol III^22^,^23^ may have been due to whether conditions in their experiments, i.e., the presence or absence of dNTPs, were suitable or not for ongoing polymerization. Moreover, while blockage of ongoing polymerization has been considered a common signal that triggers the transition from polymerization to proofreading^22^, translesion DNA synthesis^41^, or recycling the polymerase to the next Okazaki fragment^42^, we speculate that conditions suitable for ongoing polymerization may correspondingly serve as a prerequisite for inducing the transition from other functional states back to the polymerization mode. Further investigation is required to substantiate this hypothesis.

Our reconstitution of the *Mtb* DNA pol III holoenzyme and leading-strand replication *in vitro* (Fig. 1c) provides a foundation that may contribute to the development of TB drugs targeting bacterial replication. The structure of the active center of bacterial DNA pol III^43^,^44^ is very different from that of eukaryotic genome replicases^45^, making the DNA pol III system a promising target for the development of patient-friendly drugs. A number of DNA pol III holoenzyme systems from other bacteria have been reconstituted^46^,^47^,^48^ and used to screen antibacterial agents^49^,^50^. As DNA pol III is a complex macromolecular machine in which multiple subunits interact synergistically at different stages of replication^4^, screening of DNA pol III inhibitors should be based on the replication process of the complete replicase in order to identify inhibitors that not only target the active sites of individual subunits, such as the 6-anilinouracil inhibitors of the Gram-positive Pol C replicase^51^, but also those, such as bacteriophage peptides, that block intermolecular interactions at specific stages^52^. Our reconstitution of leading-strand replication by the *Mtb* DNA pol III holoenzyme provides an experimental platform that will facilitate studies on the *Mtb* DNA pol III replication mechanism and screening for effective drugs at the level of the complete replicase.

In conclusion, our reconstitution of *Mtb* DNA pol III holoenzyme leading strand replication and investigation of the physical and functional relationships between its key components, α, β_2_ and ‘ε’, has not only laid a foundation for studies on the *Mtb* DNA replication mechanism and the screening of drugs against bacterial replication, but has also provided important insights on the mechanism by which DNA pol III regulates its polymerase and exonuclease activities. It will indeed be of interest to see whether the characteristics of *Mtb* DNA pol III identified here, including the potential involvement of two proofreading subunits and a potential regulatory role played by the β_2_ clamp, do indeed affect the replicative rate and fidelity of the *Mtb* genome and influence proliferation and emergence of drug-resistance in this pathogen.

## Methods

### Protein purification

The *Mtb* DNA Pol III α and ‘ε’ subunits, clamp loading complex and single-strand binding protein (SSB) were cloned, expressed and purified in *E. coli*. A detailed description of procedures used is provided in the Supplementary Methods. Expression of the β_2_ clamp in *E. coli* was induced with 0.4 mM IPTG. It was purified as described previously^11^.

### DNA

Oligonucleotides used in biochemical assays were synthesized by BGI, Shenzhen, China, and their sequences are listed in Supplementary Table S1. Radioactive oligonucleotides were labeled with [γ-^32^P] ATP (6000 Ci/mmol) (PerkinElmer Life Sciences) using T4 polynucleotide kinase (T4 PNK) (New England Biolabs). The SSB-coated primed-M13mp18 ssDNA template used in the leading-strand replication assay was prepared by annealing a 5′-^32^P-labeled 30-mer (map positon 6852–6881) to M13mp18 ssDNA (New England Biolabs) in a 1:1 molar ratio in annealing buffer (20 mM Tris-HCl pH 7.5, 100 mM NaCl) at 94 °C for 4 min and then slowly cooling it to room temperature over 8 h. The annealed DNA mixture was then incubated with a 350-fold molar excess of SSB_4_ at 16 °C for 2 h. In exonuclease activity assays, 5′-^32^P-labeled 40-mers or 5′-^32^P-labeled 20-mers were adopted as single-strand DNA substrates, and 5′-^32^P-labeled 40-mers were annealed to the unlabeled single-stranded oligonucleotides Template-40 (40-mers) and Template-50 (50-mers) in a 1:1.2 molar ratio to produce radioactive blunt-dsDNA and 5′ overhanging-dsDNA substrates, respectively. In primer-extension assays, a 5′-^32^P-labeled 20-mer was annealed to unlabeled ssDNA Template-40 to serve as the substrate.

### DNA Pol III holoenzyme leading-strand replication assay

The holoenzyme of the *Mtb* DNA Pol III was reconstituted using proteins purified from *E. coli* (Supplementary Methods). The α, β_2_ and ‘ε’ subunits were mixed at a molecular ratio of 1:1:1 and pre-incubated at 16 °C for 2 h to fully interact with each other and the clamp loader complex (τ_3_δδ’) was purified by co-lysing cells expressing the τ, δ, and δ’ subunits. An isotope-labelled primed single-stranded phage genomic DNA (M13mp18) was used as the substrate to mimic the leading-strand in genomic replication. Standard leading-strand replication reactions contained 2.5 nM SSB-coated primed-M13mp18 ssDNA template, 1 μM αβ_2_‘ε’, 0.5 μM clamp loader (τ_3_δδ’), 0.25 mM of each dNTP, 2 mM ATP, and 10 mM MgCl_2_ in 20 mM Tris-HCl pH 7.5, containing 100 mM NaCl, 2 mM DTT, 10% (v/v) glycerol, and 50 μg/ml BSA, in a final volume of 80 μl. The reaction was started by the addition of the αβ_2_‘ε’ mixture and 10 μl aliquots were removed at each time point indicated and quenched by adding 6.6 μl loading buffer (62.5% deionized formamide, 1.14 M formaldehyde, 200 μg/ml bromphenol blue, 200 μg/ml xylene cyanole, 50 mM MOPS, 12.5 mM sodium acetate, and 100 mM EDTA pH 8.0). A control leading-strand replication reaction with the α subunit alone was performed in an identical manner, except that the DNA Pol III holoenzyme was replaced by the α subunit. One-half of the quenched reaction in each case was heated to 94 °C for 3 min and then chilled on ice immediately before loading on a 15% denaturing polyacrylamide gel containing 8 M urea.

### Exonuclease activity assays

Standard exonuclease reactions contained 5 nM radioactive DNA substrate, ‘ε’ or α subunits at the indicated concentrations and 10 mM MgCl_2_ in 20 μl of reaction buffer (20 mM Tris-HCl pH 7.5, 150 mM NaCl, 2 mM DTT, 10% (v/v) glycerol, 50 μg/ml BSA). MgCl_2_ was replaced by other divalent metal ions where indicated. Reactions were initiated upon the addition of the enzyme (‘ε’, ‘ε’^-exo^ or α) and quenched with 13.2 μl loading buffer after incubating at 37 °C for 30 min. A quarter of each quenched reaction was analyzed by 15% denaturing (8 M urea) PAGE.

### ICP-OES quantitation of metal ions

Inductively coupled plasma optical emission spectrometry (ICP-OES) was performed on a Vista MPX (VARIAN) at Tsinghua University, Beijing, China. All chemicals used were ultra-pure reagents from Sigma, St Louis, USA. Samples consisted of 3 ml of 1 mg/ml purified *Mtb* ‘ε’ subunit solution in analysis buffer (20 mM Tris-HCl pH 7.5, 100 mM NaCl, 10% (v/v) glycerol). Quantities of Mg^2+^ and Mn^2+^ bound to native ε were measured by analyzing the specific atomic emission spectra of different metal ions in continuous spectra of the samples.

### Co-immunoprecipitation (Co-IP) assays

Antibodies against the α, β and ‘ε’ subunits were prepared at the Institute of Genetics and Development Biology, Chinese Academy of Sciences, by immunizing mice with the corresponding protein. Lysate-supernatants from exponentially growing *Mtb* H37Rv cultures were prepared as described in the Supplementary Methods. Supernatants were incubated with sufficient Protein G Agarose beads (Beyotime Institute of Biotechnology) at 4 °C for 8 h, and then centrifuged at 700 g for 3 min to remove proteins non-specifically bound to the beads. The resulting supernatants were divided into four parts and respectively incubated with anti-α, anti-β, anti-‘ε’ and pre-immune sera in the presence of protein G beads at 4 °C overnight. Pre-immune serum was used as a native control to rule out false positive results. Beads were then collected by centrifugation and resuspended in 30 μl PBS buffer. 15 μl samples were analyzed by Western blotting using anti-α or anti-β serum as primary antibodies. An HRP-conjugated EasyBlot anti-Mouse IgG (Gene Tex) which specifically bound the native form of mouse IgG and decreased the interference caused by the IgG used for Co-IP was used as a secondary antibody.

### Bio-Layer Interferometry (BLI) assays

BLI assays were conducted with an Octet RED 96 System and AR2G sensors (Forte Bio). Sensors were soaked in 20 μg/ml β_2_ solution diluted by 10 mM Sodium acetate pH 4.5 (GE, Healthcare) for 10 min to coat with β_2_. Purified ‘ε’ was diluted to six different concentrations in BLI buffer (50 mM HEPES pH 7.5, 100 mM NaCl, 0.005% (v/v) Tween20) to serve as analyte samples. After equilibrating in BLI buffer for 2 min, ‘ε’ was associated with β_2_ by soaking the β_2_-bound sensors in different concentrations of ‘ε’ for 4 min, and then disassociated by soaking in BLI buffer for 8 min. Kinetic parameters of the ‘ε’-β_2_ interaction were obtained by fitting sensorgrams for different concentrations of ‘ε’. The α-‘ε’ interaction was detected in the same way as the ‘ε’-β_2_ interaction, except that 50 μg/ml α in 10 mM Sodium acetate (pH 4.0) was immobilized on the sensors.

### Surface Plasmon Resonance (SPR) assays

SPR assays were performed on a BIAcore3000 (BIAcore AB) equipped with an amino coupling CM281 chip (GE, Healthcare). Proteins used as the stationary phase in this assay were in HEPES buffer (50 mM HEPES pH 7.5, 100 mM NaCl, 10% (v/v) glycerol). 20 μg/ml purified β subunit in 10 mM Sodium acetate pH 4.0 (GE, Healthcare) was immobilized on the chip, generating about 1800 response units (Ru). A series of serially-diluted samples of α (0.01, 0.1 and 1 μM) in SPR buffer (50 mM HEPES pH 7.5, 100 mM NaCl, 0.005% (v/v) Tween20) were injected sequentially into a channel of a β_2_-coated chip at 30 μl/min for 60 s, followed by SPR buffer for 300 s to determine the value of K_D_ (α-β_2_).

### Site-directed mutagenesis

We constructed a mutant ‘ε’^-exo^ protein, in which residues Asp20, Glu22 and Asp104 involved in the putative active site of ‘ε’ were mutated into alanine. Mutations were introduced into the pET28a-SUMO/MtbdnaQ plasmid (see Supplementary Methods) via two PCR reactions catalyzed by *Pyrobest* DNA polymerase (Takara) and were confirmed by DNA sequencing. The double mutant D20A/E22A was obtained in the first PCR reaction, and the third mutation, D104A, was introduced in a second PCR reaction. Sequences of primers used in mutagenesis are shown in Supplementary Table S2. The ‘ε’^-exo^ mutant protein was expressed and purified in the same way as the wild-type ‘ε’ subunit (Supplementary Methods).

### Primer-extension assays

Primer-extension assays contained 5 nM radioactive primed dsDNA, different combinations of α, β_2_ and ‘ε’ at different concentrations, 0.25 mM of each dNTP, 10 mM MgCl_2_ in 20 μl of 20 mM Tris-HCl pH 7.5, 150 mM NaCl, 2 mM DTT, 10% (v/v) glycerol, and 50 μg/ml BSA. Protein components were mixed and pre-incubated at 16 °C for 2 h to allow time for associations to form, and then added to initiate the reaction. After incubating for 10 min at 37 °C, reactions were quenched with loading buffer (13.2 μl). A quarter of each quenched reaction was analyzed by 15% denaturing (8 M urea) PAGE.

## Additional Information

**How to cite this article**: Gu, S. *et al.* The β_2_ clamp in the *Mycobacterium tuberculosis* DNA polymerase III αβ_2_ε replicase promotes polymerization and reduces exonuclease activity. *Sci. Rep.* **6**, 18418; doi: 10.1038/srep18418 (2016).

## Supplementary Material

Supplementary Information
