# Supplementary material for: The β2 clamp in the Mycobacterium tuberculosis DNA polymerase III αβ2ε replicase promotes polymerization and reduces exonuclease activity
Source: Sci Rep. 2016 Jan 29;6:18418. doi: 10.1038/srep18418 (PMC4731781; doi:10.1038/srep18418)
Supplement: Supplementary Information [file srep18418-s1.doc]

**SUPPLEMENTARY INFORMATION:**

**The β_2_ clamp in the *Mycobacterium tuberculosis* DNA polymerase III αβ_2_ε replicase promotes polymerization and reduces exonuclease activity**

Shoujin Gu^1,6,7^, Wenjuan Li^1,7^, Hongtai Zhang^1^, Joy Fleming^1^, Weiqiang Yang^2^, Shihua Wang^2^, Wenjing Wei^1^, Jie Zhou^3^, Guofeng Zhu^4^, Jiaoyu Deng^5^, Jian Hou^1^, Ying Zhou^1^, Shiqiang Lin^1,2^, Xian-En Zhang^1,*^ and Lijun Bi^1,*^.

^1^ Key Laboratory of RNA Biology & National Laboratory of Biomacromolecules, Institute of Biophysics, Chinese Academy of Sciences, Beijing 100101, China

^2^ School of Life Sciences, Fujian Agriculture and Forestry University, Fuzhou 350002, China

^3^ The Fourth People’s Hospital, Foshan 528000, China

^4^ Shanghai Municipal Center for Disease Control and Prevention, Shanghai 200336, China

^5^ State Key Laboratory of Virology, Wuhan Institute of Virology, Chinese Academy of Sciences, Wuhan 430071, China

^6^ University of Chinese Academy of Sciences, Beijing 100049, China

^7^ These authors contributed equally to this study

* To whom correspondence should be addressed. Lijun Bi: Tel: +86-10-64888464; Fax: +86-10-64871293; Email: [blj@ibp.ac.cn](mailto:blj@ibp.ac.cn). Correspondence may also be addressed to: Xian-En Zhang: Tel: +86 10 64888262; Fax: +86-10-64871293; E-mail: [zhangxe@sun5.ibp.ac.cn](mailto:zhangxe@sun5.ibp.ac.cn)

**SUPPLEMENTARY MATERIALS AND METHODS**

**Purification of *Mtb* DNA Pol III subunits**

Genes encoding each of the subunits of DNA pol III were amplified by PCR from *Mtb* genomic DNA using the *KOD-plus* DNA polymerase from Toyobo, Osaka, Japan. Primers used in this study are listed in Supplementary Table 3S, and were synthesized by BGI, Shenzhen, China. All DNA-modifying enzymes, including restriction endonucleases and T4 DNA ligase, were purchased from New England Biolabs, Ipswich, MA. Recombinant plasmids were confirmed by sequencing. The corresponding proteins were purified according to the procedures outlined below and their identity was confirmed by means of peptide mass fingerprinting (PMF) using matrix-assisted laser desorption/ionization time-of-flight mass spectrometry (MALDI-TOF MS).

**α subunit (*dnaE1*; Rv1547)**

The PCR product of Rv1547 was ligated into the pET28a-MBP vector containing a MBP-Tag ([maltose-binding protein](http://www.iciba.com/maltose-binding_protein), 40kDa) to create the pET28a-MBP/MtbdnaE1 plasmid. The recombinant plasmid was then transformed into *E. coli* BL21(DE3) cells (Novagen). Cells were grown in LB medium at 37°C until the OD_600_ reached 0.6. The culture medium was then cooled to 16°C, and 0.4 mM isopropyl β-D-1-thiogalactopyranoside (IPTG) was added to induce protein expression. Cells were harvested by centrifugation after 16 h of induction. Cell pellets were resuspended with precooled buffer A (20 mM Tris-HCl pH 8.0, 500 mM NaCl, 5 mM [dithiothreitol](http://www.iciba.com/dithiothreitol) (DTT), 2 mM EDTA, 10% glycerol, 1 mM phenylmethyl sulfonylfluoride (PMSF)), sonicated in an ice-water waterbath, then centrifuged at 18,000 rpm for 30 min at 4°C. Supernatants were incubated with Amylose Resin (New England Biolabs) for 2 h at 4°C, and then the resin was eluted with at least a 5-fold volume of buffer A. The target protein was eluted with buffer A containing 10 mM maltose. After concentration, the protein, in buffer A, was applied to a Superdex^TM^ 200 10/300 GL column (GE Healthcare), then dialyzed against buffer B (20 mM Tris-HCl pH 7.5, 100 mM NaCl, 2 mM [dithiothreitol](http://www.iciba.com/dithiothreitol) (DTT), 0.5 mM EDTA, 10% glycerol). The MBP-tag was cleaved by incubating for 16 h at 4°C in the presence of TEV protease (tobacco etch virus protease, 27kDa) (enzyme:protein = 1:30 w/w) which was added twice, half at the beginning and the other half in the middle of the incubation. The MBP tag was removed from the fusion protein; only two residues (GH) were left attached to the N-terminus of the MtbDnaE1 protein.The protein was dialyzed against buffer C (20 mM Tris-HCl pH 7.5, 100 mM NaCl, 10% glycerol) and further purified by a second round of size exclusion chromatography to remove any free MBP-tag. Fractions containing the target protein were pooled and concentrated before aliquoting and storing at -80°C.

**'ε' subunit (*dnaQ*; Rv3711c)**

The PCR product of Rv3711c was cloned into a pET28a-SUMO expression vector creating the pET28a-SUMO/MtbdnaQ plasmid. Protein expression and cell lysis procedures were the same as those used to purify the α subunit, except that buffer D (20 mM Tris-HCl pH 7.5, 500 mM NaCl, 5 mM imidazole, 10% glycerol) was used in lysis. Supernatants were incubated with Ni^2+^-chelating Sepharose^TM^ Fast Flow (GE Healthcare) for 3 h at 4°C, and eluted using an imidazole gradient of 30, 60, 100 and 500 mM imidazole, the target protein eluting at 500 mM imidazole. The protein was applied to a Superdex^TM^ 200 10/300 GL column (GE Healthcare) in buffer D without imidazole. The target protein was collected and dialyzed against buffer E (20 mM Tris-HCl pH 8.5, 20 mM NaCl). The dialyzed protein was further loaded onto a 5ml HiTrap Q Sepharose FF (GE Healthcare), and was eluted with a linear 100 ml gradient of 0-1000 mM NaCl in buffer E. Fractions containing the target protein were pooled and dialyzed against buffer C. The dialyzed protein was incubated with Ulp1 protease (enzyme:protein = 1:50 w/w) at 4°C for 8 h to cleave the N-terminal His_6_-SUMO tag from the DnaQ protein. The resulting protein was concentrated and further purified by applying to a Superdex 75 10/300 GL column (GE Healthcare) in buffer C. The final preparation of the 'ε' subunit (MtbDnaQ) was aliquoted and stored at -80°C.

**Clamp loader complex (τ_3_δδ’) (*dnaZX*, *holA* and *holB*; Rv3721c, Rv2413c and Rv3644c)**

The PCR product of Rv3721c was cloned into the pACYCDuet-1 vector (Novagen), generating a pACYCDuet-1/MtbdnaZX plasmid. At the same time, the PCR products of the Rv2413c and Rv3644c were inserted into pET28a vectors (Novagen) to produce pET28a/MtbholA and pET28a/MtbholB, respectively. The three recombinant plasmids were then respectively transformed into E. coli BL21 (DE3) to express the τ, δ and δ’ subunits.

Cells expressing these three subunits were mixed according to their wet weight before being lysed in buffer D containing 2 mM β-mercaptoethanol. Cells expressing δ and those expressing δ’ were in a >7-fold excess to those expressing τ, ensuring that τ would be entirely saturated by δ and δ’ in the supernatant after lysis. The clamp loader was purified by Ni^2+^-IMAC utilising the N-terminal His_6_-tag of each protein. After elution from the Ni^2+^-NTA resin, the concentration of β-mercaptoethanol and EDTA of the pooled fractions containing the eluted proteins was adjusted to 10 mM and 2 mM, respectively. The resulting proteins were applied to a Superdex^TM^ 200 10/300 GL column (GE Healthcare) in buffer F (20 mM Tris-HCl pH 7.5, 500 mM NaCl, 10 mM β-mercaptoethanol, 2 mM EDTA and 10% glycerol) to remove unbound δ and δ’. The resulting complex was dialyzed against buffer G (20 mM Tris-HCl pH 7.5, 100 mM NaCl, 2 mM β-mercaptoethanol, 0.5 mM EDTA and 10% glycerol) and then analyzed on a 12% SDS-polyacrylamide gel. After staining with Coomassie Blue R-250, protein band intensities were measured by ImageJ to estimate the subunit ratio. The final preparation of the *Mtb* clamp loader complex was aliquoted and stored at -80°C.

**SSB (*ssb*, Rv0054)**

The PCR product of Rv0054 was cloned into the pET28a vector to produce the pET28a/Mtbssb plasmid. Procedures for protein expression, cell lysis and Ni^2+^-IMAC purification were performed as described above for the purification of DnaQ. The target protein was dialyzed against buffer E and then subjected to anion-exchange chromatography using a 5 ml HiTrap Q Sepharose FF column (GE, Healthcare). Elution of the protein began at 320 mM NaCl in a linear gradient of 0-1000 mM NaCl in buffer E. The eluted protein was dialyzed against buffer C and then subjected to further size-exclusion chromatography using a Superdex^TM^ 200 10/300 GL column (GE, Healthcare). The final preparation of the SSB protein (MtbSSB) was aliquoted and stored at -80°C.

**Preparation of *Mtb* H37Rv cell lysate supernatants**

*Mtb* H37Rv cells were cultured in 2L of 7H9 media (Becton, Dickinson and Company) at 37°C and collected by centrifugation at 5000g for 15 min at 4°C when the culture had reached the exponential phase. The cell pellet was resuspended in 25 ml precooled protease inhibitor-containing PBS buffer containing one cOmplete, Mini, EDTA-free tablet (Roche) per 10 ml PBS buffer, and divided into 2 ml lysing matrix B tubes containing 0.1 mm silica beads (MP Bio Science Ltd) (1 ml suspension per tube). Cells were lysed with a FastPrep-24 (MP Bio Science Ltd). After centrifugation at 15000 rpm for 15 min at 4°C, the supernatant was collected and passed through a 0.22 μm sterile filter to filter out any remaining live cells. All experiments involving viable *Mtb* were performed in a BSL-3 laboratory at Beijing Tuberculosis and Thoracic Tumor Research Institute.

**Isothermal Titration Calorimetry**

The binding affinity of the MTB DNA pol III 'ε' and α subunits was measured by isothermal titration calorimetry on a MicroCal iTC200 (Marlvern). Proteins were first thoroughly dialyzed against buffer containing 50 mM HEPES, pH 7.5, 100 mM NaCl and 10% (v/v) glycerol, then degassed to remove air bubbles. The 'ε' subunit was adjusted to a final concentration of 11 μM and loaded into the injection syringe, while the sample cell was loaded with a 200 μM solution of the α subunit. Titration reactions were performed with 20 injections, all in 2 μl volume, with constant stirring at 600 rpm at 25°C. Injections were at intervals of 150 s, and the duration of each injection was 4 s. The experiment was performed in triplicate.

**Sequence alignment and secondary structure prediction**

Genes encoding the subunits of DNA pol III were identified by searching bacterial genomes using the protein sequences of the corresponding *E. coli* genes with protein BLAST (<http://blast.ncbi.nlm.nih.gov/Blast.cgi>) and default parameters. Multiple sequence alignments of the α subunits and the ε subunits form different bacteria were carried out with Clustal Ω (<http://www.ebi.ac.uk/Tools/msa/clustalo/>). PSIPRED and DISOPRED were used to predict the secondary structures and the disordered regions of the ε subunits from *E. coli* and *Mtb*. The molecular weights (Mw) of the proteins were calculated with the “compute pI/Mw” tool on the ExPASy server .

**SUPPLEMENTARY TABLES**

**Table S1. Oligonucleotides used as DNA substrates.**

| Primer | Sequence |
| --- | --- |
| 20-mer | 5’-TGAACGGTAATCGTAAAACT-3’ |
| 30-mer | 5’-TGAACGGTAATCGTAAAACTAGCATGTCAA-3’ |
| 40-mer | 5’-TGAACGGTAATCGTAAAACTAGCATGTCAATCATATGTAC-3’ |
| Template-40 | 5’-GTACATATGATTGACATGCTAGTTTTACGATTACCGTTCA-3’ |
| Template-50 | 5’-ATCAACCGGGGTACATATGATTGACATGCTAGTTTTACGATTACCGTTCA-3’ |

**Table S2. Primers used in site-directed mutagenesis of the *Mtb* 'ε' subunit.**

| Primer | Sequence |
| --- | --- |
| MutD20A/E22A -sense | 5’-GATCGGGGTTGGGCCGTCATCGCTGTCGCGACCTCGGGCTTTCGGCCGGGC-3’ |
| MutD20A/E22A-antisense | 5’-GCCCGGCCGAAAGCCCGAGGTCGCGACAGCGATGACGGCCCAACCCCGATC-3’ |
| MutD104A-sense | 5’-CGCGCACAATGTCGCGTTCGCTTATGCGTTTCTCGCTGCGGA-3’ |
| MutD104A-antisense | 5’-TCCGCAGCGAGAAACGCATAAGCGAACGCGACATTGTGCGCG-3’ |

**Table S3. Primers used in standard gene amplification**

| Primer | Sequence | Restriction  endonuclease |
| --- | --- | --- |
| dnaE1-sense | 5’-GGAATTCCATATGAGCGGTTCATCTGCGG-3’ (29-mer) | NdeI |
| dnaE1-antisense | 5’-CCCAAGCTTCTAACTCCCCAGACATCCAG-3’ (29-mer) | HindIII |
| dnaQ-sense | 5’-CGCGGATCCATGAGCCACACCTGGGGAC-3’ (28-mer) | BamHI |
| dnaQ-antisense | 5’-CGGTCAAGCTTTCAGAACAACGCTAACTGCTT-3’ (32-mer) | HindIII |
| dnaZX-sense | 5’-CGCGGATCCGATGGCTCTCTA-3’ (29-mer) | BamHI |
| dnaZX-antisense | 5’-CGAGCTCTAGGCGTTGTCTAT-3’ (29-mer) | SacI |
| holA-sense | 5’-GGAATTCCATATGCACCTGGTCCTGGGAGACG-3’ (32-mer) | NdeI |
| holA -antisense | 5’-TTATACTCGAGTCATCGGCCGCGGTCGGC-3’ (29-mer) | XhoI |
| holB-sense | 5’-GGAATTCCATATGCCGATGATGTCCGGGGTGT-3’ (32-mer) | NdeI |
| holB -antisense | 5’-TTATACTCGAGCTACCGCAGTTCCTGGCC-3’ (29-mer) | XhoI |
| SSB-sense | 5’-GGAATTCCATATGGTGGCTGGTGACACCACCA-3’ (32-mer) | NdeI |
| SSB-antisense | 5’-CTTGCGAATTCGTGGCTGGTGACACCACCA-3’ (30-mer) | EcoRI |

**SUPPLEMENTARY FIGURES**
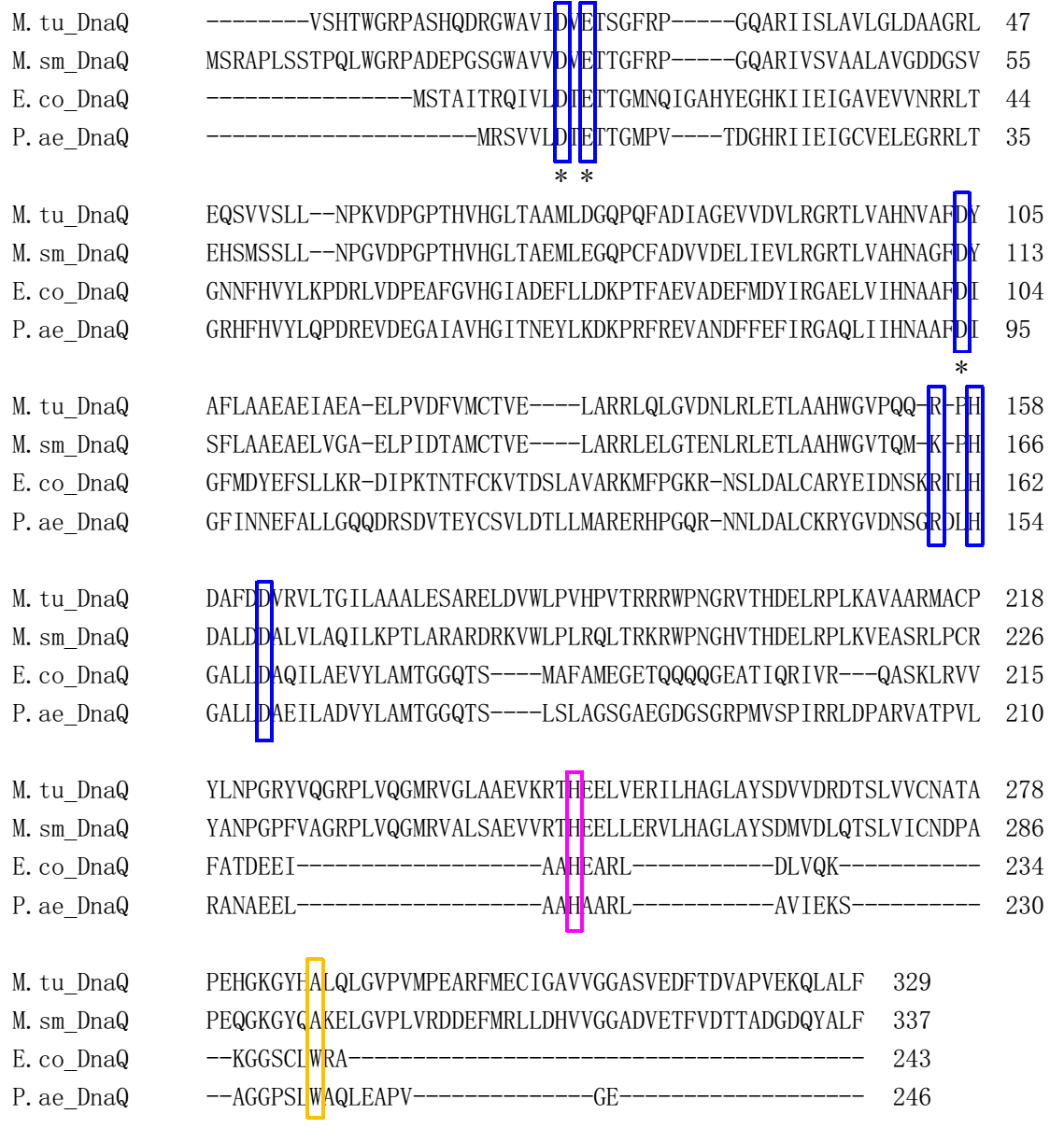


**Supplementary Figure S1.** Sequence alignment of the ε subunits from *M. tuberculosis*, *M. smegmatis, E. coli* and *P. aeruginosa*. The ε subunits of *M. tuberculosis*, *M. smegmatis, E. coli* and *P. aeruginosa* were aligned using Clustal Ω. The six conserved residues (D20, E22, D104, R155, H158 and D163) in cyan rectangles are predicted to be the ‘DEDDh’ exonuclease active residues of the *M. tuberculosis* 'ε' subunit. Three of these residues (indicated with asterisks) were substituted by Ala to obtain a mutant ('ε'^exo-^) with defective exonuclease activity. The residues probably involved in interacting with the α subunit (EcoDnaQ His225; MtuDnaQ His247; MsmDnaQ His255; PaeDnaQ His220 and EcoDnaQ Trp241; PaeDnaQ Trp237), are indicated by magenta and gold rectangles, respectively . Amino acid residue numbers of the ε subunit are indicated to the right of the sequence.

**
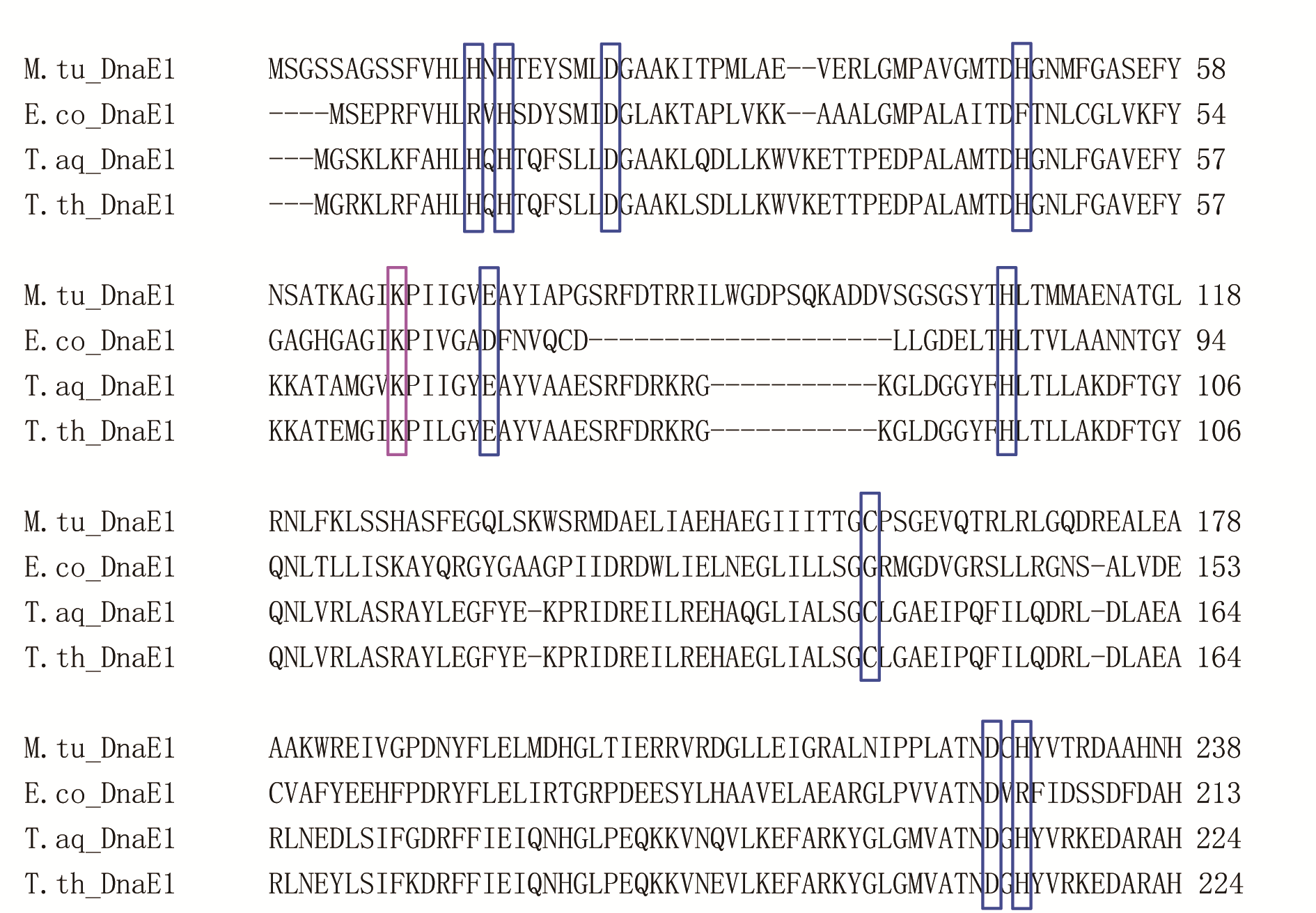
Supplementary Figure S2.** Sequence alignment of the PHP metal-binding site in the DNA pol lII α subunit from *M. tuberculosis*, *E. coli*, *T. aquaticus* and *T. thermophilus* using the Clustal Ω alignment tool. The putative metal-binding site of the α subunit PHP domain (*M. tuberculosis* DnaE1: H14, H16, D23, H48, E73, H107, C158, D226 and H228) in blue rectangles is highly conserved in most bacteria, except in *E. coli* which belongs to the *Proteobacteria*. The residue probably involved in interacting with the ε subunit (identical in all four species) is indicated with a magenta rectangle (MtuDnaE1: Lys67; EcoDnaE1: Lys63; TaqDnaE1: Lys66 and TthDnaE1: Lys66) . Amino acid residue numbers of the α subunit are indicated to the right of the sequence.

**a**


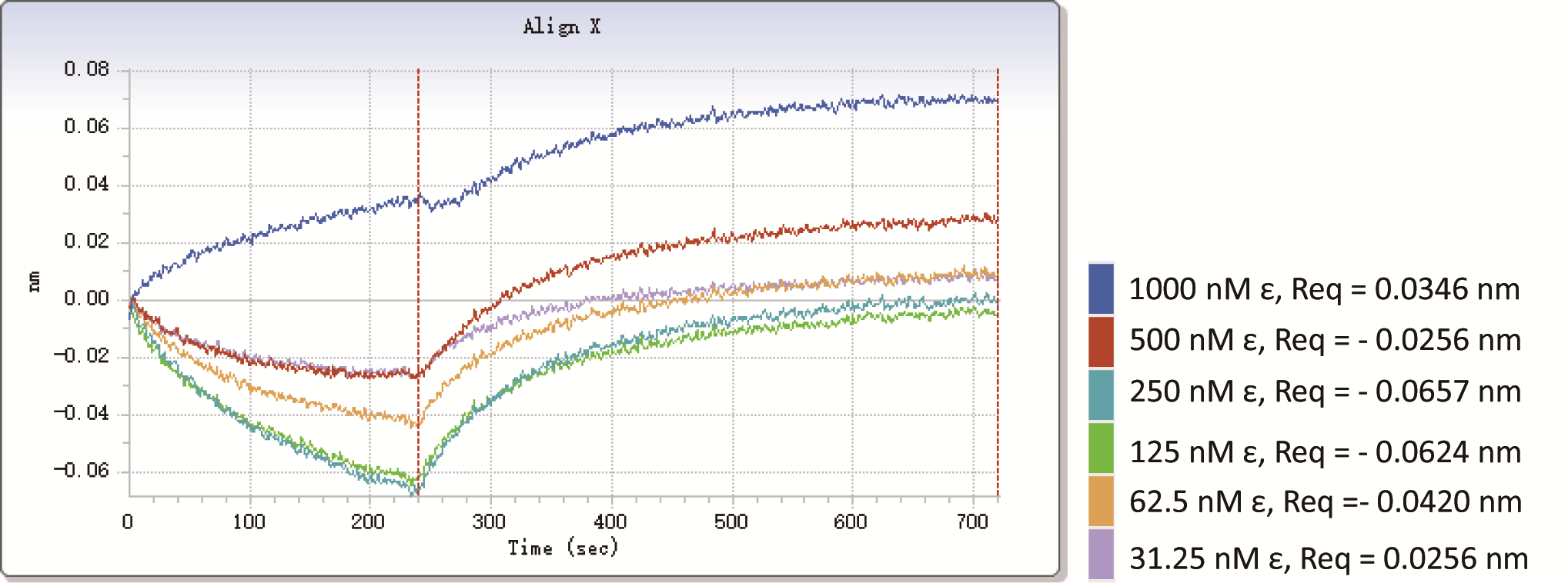
**b**

**
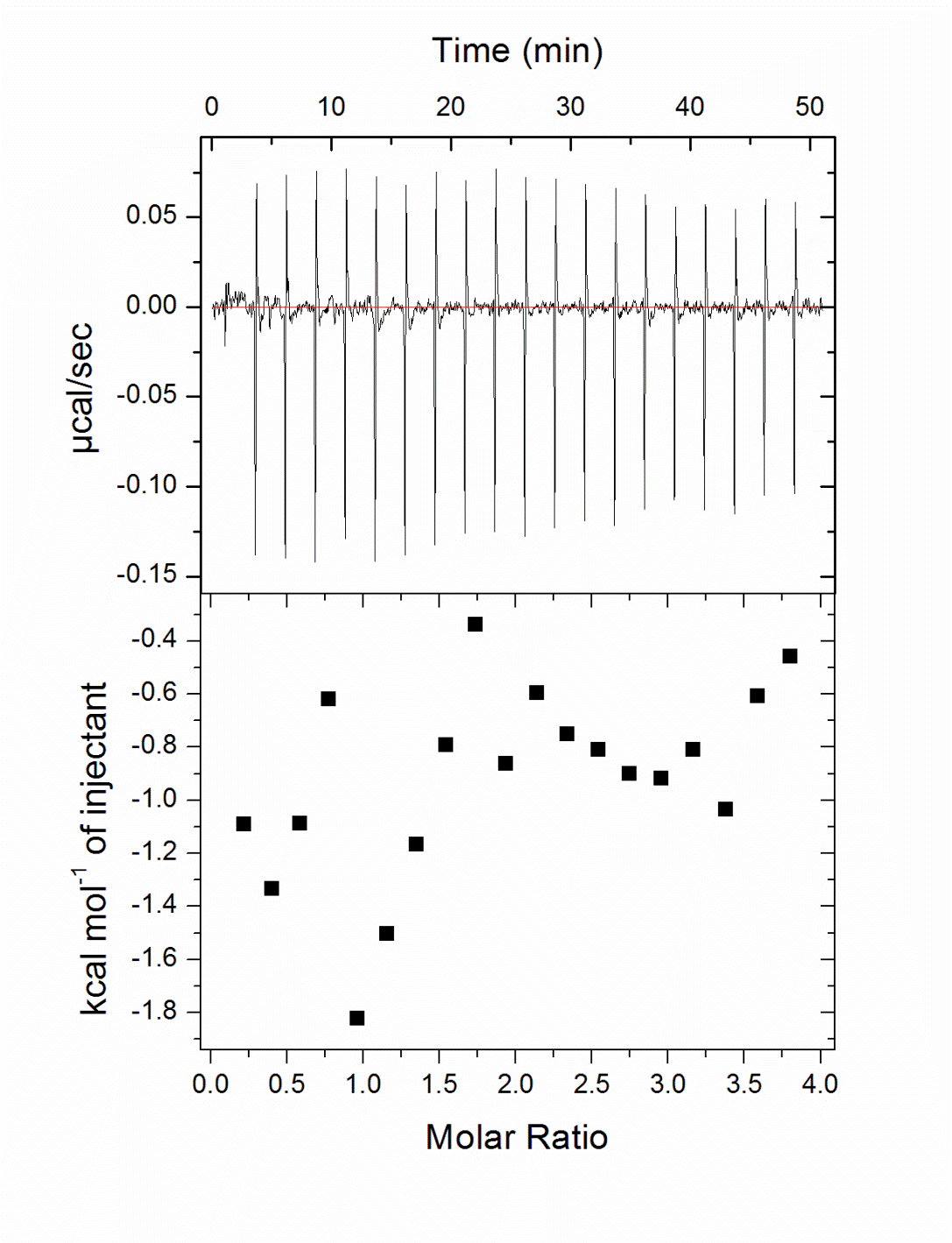
**

**Supplementary Figure S3.** **(a)** Detection of interactions between the α and 'ε' subunits of *Mtb* DNA pol III by Bio-Layer Interferometry. The α subunit was used as the protein in the stationary phase, and six concentrations of the 'ε' subunit (31.25, 62.5, 125, 250, 500 and 1000 nM) were adopted as the analyte samples in the mobile phase. The 'ε' subunit appears not to directly interact with the α subunit under the conditions used here. The slight rise in the curve of 1000 nM 'ε' in the association period (first 240 s) resulted from nonspecific binding between the 'ε' subunit and the sensors. **(b)** Detection of interactions between the α and 'ε' subunits of *Mtb* DNA pol III by isothermal titration calorimetry. A 11 μM solution of the α subunit was loaded into the sample cell and the 'ε' subunit at a concentration of 200 μM served as the titrant. No specific energy emission was detected indicating that the 'ε' subunit does not bind directly to the α subunit under these experimental conditions.


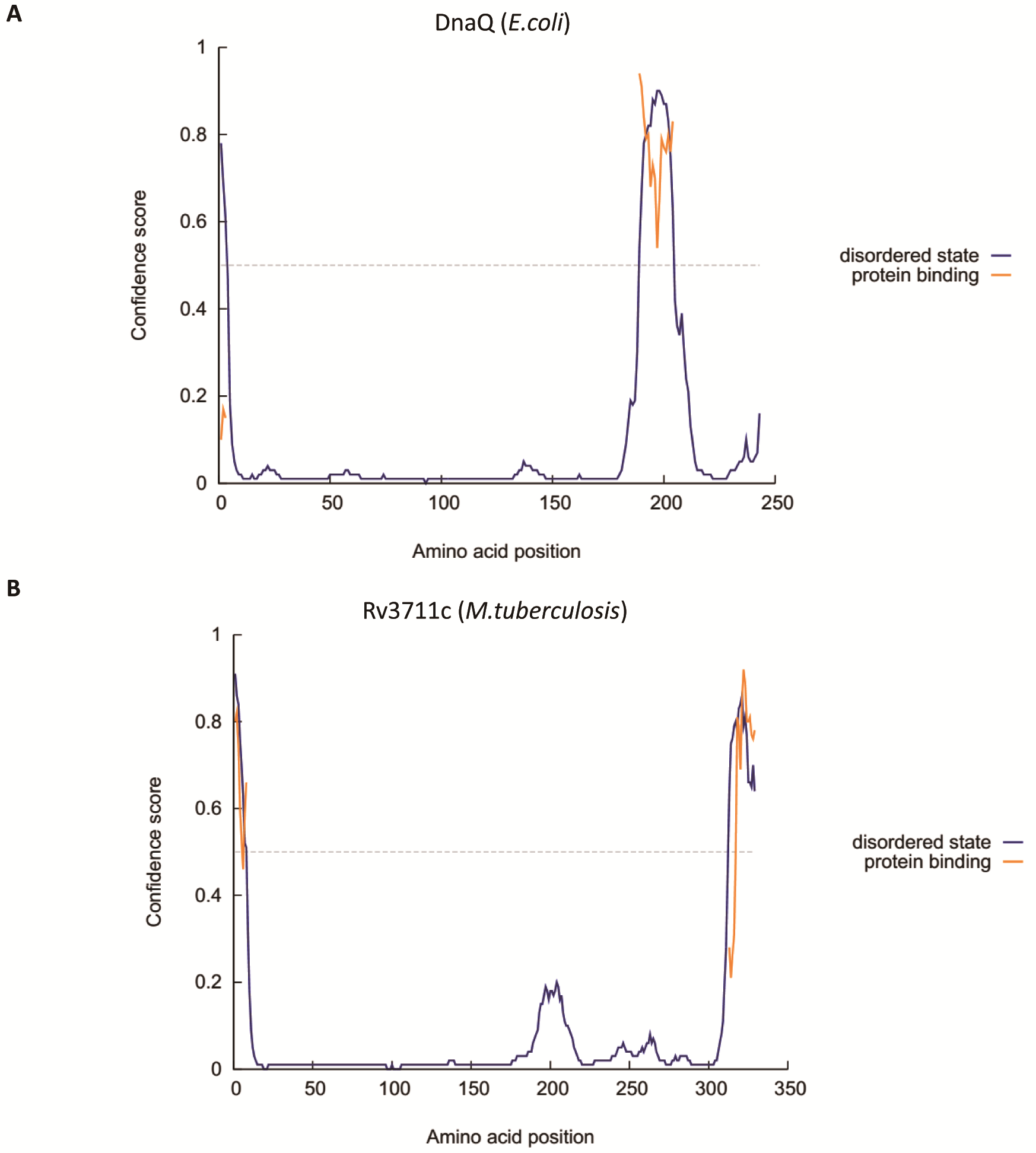


**Supplementary Figure S4.** Intrinsic disorder profiles of the *E. coli* ε subunit and its *Mtb* homologue. The sequences of *E. coli* DnaQ (**A**) and *Mtb* Rv3711c (**B**) were analyzed using the DISOPRED tool. Amino acids are considered disordered or capable of binding proteins when the line is above the grey dashed line, that is, the confidence score is higher than 0.5. Rv3711c has a disordered potential protein-binding region in its C-terminal segment. The N-terminal 180 residues are well-structured, similar to those in *E. coli* DnaQ.

**Supplementary References**

1. Cole, S.T., Brosch, R., Parkhill, J., Garnier, T., Churcher, C., Harris, D., Gordon, S.V., Eiglmeier, K., Gas, S., Barry, C.E., 3rd *et al.* (1998) Deciphering the biology of *Mycobacterium tuberculosis* from the complete genome sequence. *Nature*, **393**, 537-544.

2. Schneider, C.A., Rasband, W.S. and Eliceiri, K.W. (2012) NIH Image to ImageJ: 25 years of image analysis. *Nat. Methods*, **9**, 671-675.

3. Jones, D.T. (1999) Protein secondary structure prediction based on position-specific scoring matrices. *J. Mol. Biol.*, **292**, 195-202.

4. Jones, D.T. and Ward, J.J. (2003) Prediction of disordered regions in proteins from position specific score matrices. *Proteins*, **53 Suppl 6**, 573-578.

5. Wilkins, M.R., Gasteiger, E., Bairoch, A., Sanchez, J.C., Williams, K.L., Appel, R.D. and Hochstrasser, D.F. (1999) Protein identification and analysis tools in the ExPASy server. *Methods Mol. Biol.*, **112**, 531-552.

6. Timinskas, K., Balvociute, M., Timinskas, A. and Venclovas, C. (2014) Comprehensive analysis of DNA polymerase III alpha subunits and their homologs in bacterial genomes. *Nucleic Acids Res.*, **42**, 1393-1413.
